# Recent Research on *Fusarium* Mycotoxins in Maize—A Review

**DOI:** 10.3390/foods11213465

**Published:** 2022-11-01

**Authors:** Marcin Bryła, Adam Pierzgalski, Agnieszka Zapaśnik, Pascaline Aimee Uwineza, Edyta Ksieniewicz-Woźniak, Marta Modrzewska, Agnieszka Waśkiewicz

**Affiliations:** 1 Department of Food Safety and Chemical Analysis, Waclaw Dabrowski Institute of Agricultural and Food Biotechnology—State Research Institute, 02-532 Warsaw, Poland; 2 Department of Microbiology, Waclaw Dabrowski Institute of Agricultural and Food Biotechnology—State Research Institute, Rakowiecka 36, 02-532 Warsaw, Poland; 3 Department of Chemistry, Poznań University of Life Sciences, Wojska Polskiego 75, 60-625 Poznań, Poland

**Keywords:** mycotoxins, classification, *Fusarium*, maize, occurrence, biological methods, toxicology, detoxification, modified mycotoxins

## Abstract

Maize (*Zea mays* L.) is one of the most susceptible crops to pathogenic fungal infections, and in particular to the *Fusarium* species. Secondary metabolites of *Fusarium* spp.—mycotoxins are not only phytotoxic, but also harmful to humans and animals. They can cause acute or chronic diseases with various toxic effects. The European Union member states apply standards and legal regulations on the permissible levels of mycotoxins in food and feed. This review summarises the most recent knowledge on the occurrence of toxic secondary metabolites of *Fusarium* in maize, taking into account modified forms of mycotoxins, the progress in research related to the health effects of consuming food or feed contaminated with mycotoxins, and also the development of biological methods for limiting and/or eliminating the presence of the same in the food chain and in compound feed.

## 1. Introduction

**Figure 1:**
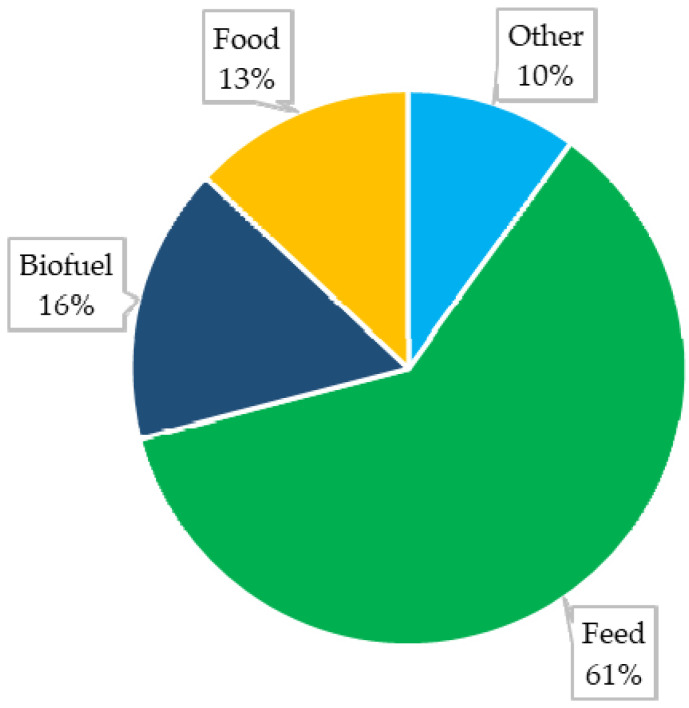
Global use of maize [2].

Maize (*Zea mays* L.) is an important—and in some countries even the most important—source of nutrition for humans and animals [1]. An increasing interest in this raw material has also been observed in various industries (Figure 1). It is estimated that as of 2022 the global production of maize will amount to approximately 1173.3 Mt from a surface area of approximately 200.4 Mha [2]. Despite a high yield potential, maize is characterised by high susceptibility to various diseases, which can lead to decreased yields and reduced kernel quality [3].

A decade ago, the *Fusarium* fungi were named as one of the ten most important pathogenic fungi in the world which affect plants, in particular cereals [4]. This genus includes a large number of species that vary morphologically and phylogenetically [5]. They are epiphytic and endophytic organisms and are part of maize’s pathogenic microbiota [6]. *Fusarium* can damage the plant by colonising the xylem and developing mycelium in the root, thus causing vascular obstruction and hindering the normal transport of water to the aerial parts of the plant.

The *Fusarium* fungi are also responsible for the biosynthesis of toxic secondary metabolites referred to as mycotoxins. Those compounds can weaken the infected plant’s immune system and inhibit cell proliferation and protein synthesis [7]. There are three main causes of *Fusarium* infection and mycotoxin biosynthesis in maize: genetic predispositions of maize varieties, manufacturing practices, and climatic conditions; with the latter appearing to be crucial. Genetic factors contribute to the diversification of vulnerability to fungal pathogens and mycotoxin contamination between various species of crops. One example is Bt maize (genetically modified for the purpose of expression of insecticidal proteins originating from *Bacillus thuringiensis*), characterised by increased resistance to *Fusarium* infection as compared to traditional maize [7,8]. Improper agricultural and manufacturing practices increase the risk of kernel infection already during the growing season, as well as during harvest, storage, transport, and processing of the raw material [7]. However, the most crucial factors are climate conditions, since the development of toxicogenic *Fusarium* species can depend, to a large extent, on the geographical location of the fields, and particularly on the temperature and humidity typical to the region [9].

Climate change increasingly disturbs weather patterns associated with the distribution of precipitation and temperature fluctuations. Extreme weather and reduced annual environmental variability can cause abrupt biotic and abiotic stress in plants. It is believed that this can have a significant impact on the course of *Fusarium* infection in plants and on the species structure of this pathogen, the host plant, and the host–pathogen interaction [5,10].

Maize is one of the most susceptible crops to pathogenic fungal infections, and in particular to the *Fusarium* fungi. Mycotoxins produced by those fungi are not only phytotoxic, but also harmful to humans and animals. They can cause acute or chronic diseases and have carcinogenic, teratogenic, immunosuppressive, or estrogenic effects [11]. To ensure food and feed safety, the EU member states apply standards and legal regulations on the permissible levels of those compounds [12].

This article summarises the most recent knowledge on: (i) the incidence of toxic fungal metabolites of *Fusarium* in maize, (ii) developments in the identification of health-related effects of ingesting food or feed contaminated with mycotoxins, and (iii) biological methods for limiting and/or eliminating the presence of the same in the food chain and in compound feed.

## 2. Classification

The most common classification of mycotoxins found in cereal grains is based on the close link between these substances and the fungal species responsible for their biosynthesis and, more broadly, the diseases they cause in maize plants. The presence of *Fusarium* fungi can cause various types of rot, including *Fusarium* ear rot (FER) and *Gibberella* ear rot (GER) [13]. The species composition of pathogenic fungi responsible for the development of these diseases in plants can change over the years and depends on geographical factors and agrotechnical conditions [14]. However, the most common pathogens which cause FER are *F. verticillioides*, *F. proliferatum*, and occasionally *F. subglutinans* [15]. The dynamics of plant colonisation by fungi responsible for the development of FER is strongly linked to environmental factors. It should be taken into account that while *F. verticillioides* is characterised by an exceptional capability to adapt to high temperatures (approx. 30 °C), *F. proliferatum* and *F. subglutinans* require lower temperatures to grow [16]. In their recent studies, Simões et al. [17] described *F. andiyazi* as one of the factors responsible for FER in maize in Europe [17]. On the other hand, GER was most often caused by *F. graminearum*, *F. culmorum*, and *F. avenaceum*. Other species, including *F. equiseti*, *F. poae*, *F. sporotrichioides*, *F. acuminatum*, *F. semitectum*, *F. solani*, and *F. temperatum* were also isolated from infected maize ears [18,19]. In these cases, the conditions which promoted the development of infection were temperatures of 24–26 °C and humidity exceeding 85% [20]. In recent years, an upward trend was observed in the incidence of certain *Fusarium* species in maize kernels in various countries. This trend is likely associated with the change in climatic conditions and the agronomic practices used [21]. Crop rotation (wheat-maize) and the presence of after-harvest residue on the soil’s surface contribute to the preservation of fungal pathogens, which increases the risk of FER and GER [22].

It has long been known that *F. verticillioides* and *F. proliferatum* biosynthesise fumonisins, which are one of the most common *Fusarium* toxins found in maize kernels. They were first isolated from the *F. verticillioides* strain and subsequently identified in *F. proliferatum* cultures and in several less known *Fusarium* species. Those compounds belong to a small group of toxins characterised by an aliphatic structure, which have been divided into four series: A, B, C and P, of which the B series is dominant. The structure of B-series fumonisins is based on a 20-carbon backbone, with molecules of 1,2,3-tricarballylic acid bonded at C14 and C15, and an amino group bonded at C2. The location and number of hydroxyl groups within the molecule determine the type of toxin [23,24,25,26,27].

Another group of toxins most often identified in maize kernels are trichothecenes. Trichothecenes are sesquiterpenoid mycotoxins. The common structural feature of these compounds is based on the 12,13-epoxytrichothecenem skeleton. Generally, there are four recognised types of trichothecenes, i.e., A, B, C, and D, whereas C and D are not produced by *Fusarium* species [28]. The diversity of the trichothecene family results from the different number of hydroxyl groups located at different positions in the basic trichothecene nucleus. Type A trichothecenes include: T-2 toxin (T-2) and HT-2 toxin (HT-2), neosolaniol (NEO), diacetoxyscirpenol (DAS), monoacetoxyscirpenol (MAS), verrucarol (VER), scirpentriol (SCP), and their derivatives. T-2 and HT-2 toxins are produced primarily by *F. sporotrichioides*, *F. acuminatum*, and *F. poae* strains, while *F. poae*, *F. equiseti*, *F. sambucinum*, and *F. sporotrichioides* strains are responsible for the biosynthesis of DAS and MAS. NEO is particularly characteristic of *F. sporotrichioides*, *F. poae*, and *F. acuminatum* strains. Type B trichothecenes are represented by deoxynivalenol (DON) and its acetylated derivatives, i.e., 3-acetyldeoxynivalenol (3-AcDON) and 15-acetyldeoxynivalenol (15-AcDON), nivalenol (NIV) and fusarenone X (FUS-X). Those toxins are produced primarily by *F. culmorum* and *F. graminearum* strains [29].

Zearalenone (ZEN) contains a resorcinol moiety fused to a 14-membered macrocyclic lactone. Similar to type B trichothecenes, ZEN can be biosynthesised by *F. culmorum* and *F. graminearum*, which is why it is often detected in combination with other fusariotoxins [30]. The biosynthesis of this toxin by fungal pathogens takes place primarily during the plant’s growth, but can also occur as a result of improper crop storage. The major issue related to ZEN exposure stems from the fact that it is one of the strongest non-steroidal compounds among naturally occurring oestrogens. ZEN opens the group of five primary compounds, the biosynthesis directions of which may vary. In addition to ZEN, this family of compounds also includes α-zearalenol (α-ZOL), β-zearalenol (β-ZOL), α-zearalanol (α-ZAL), β-zearalanol (β-ZAL), and zearalanon (ZAN) [31]. The classification of selected mycotoxins based on the pathogen is presented in Table 1.

Previous findings concerning the incidence of mycotoxins and the processes they undergo in host organisms inspired researchers to create an informal classification of those compounds. The first group includes compounds which originate in fungal metabolic pathways and are commonly referred to as “free” mycotoxins [26,40]. A classic example of “free” secondary fungal metabolites found in maize are the compounds whose concentration in food and feed is regulated by EU legislation and standards established for the countries outside the EU [41,42,43,44]. They include fumonisins, DON, ZEN and HT-2, and T-2 toxins (the characteristics of those substances were presented above).

Mycotoxins covered by standards and legal regulations are a small group; in addition, maize kernels may be affected by other compounds produced directly by fungi, such as trichothecenes (NIV, FUS-X, 3- and 15-AcDON, MAS, DAS, NEO), beauvericin (BEA), enniatins (ENNs), ZEN derivatives, and others [45].

Secondary metabolites, produced by the *Fusarium* fungi, can undergo transformation in a crop. Altered structures of mycotoxins constitute a part of “modified mycotoxins”, which have been presented in detail by Rychlik et al. [40]. Modified mycotoxins are compounds that are normally not detected during routine mycotoxin analysis. They can be biosynthesized directly by fungi or generated by the defense mechanism of a plant infected with a pathogen. Sometimes these compounds can be formed during technological processes used during food production. This classification of mycotoxins is not strict, since certain modified mycotoxins can be biosynthesised both by fungi and in the plants within metabolic pathways (e.g., α- and β-ZOL from ZEN). Furthermore, recent research confirmed that ZEN-14S can be biosynthesised directly by *Fusarium*, concurrently with ZEN, which is likely related to the stress reaction of the pathogen to the accumulation of ZEN in the substrate [46,47]. Another interesting issue is the presence of so-called “hidden mycotoxins” in maize kernels [48]. They are part of a wider group of modified mycotoxins. It is believed that fumonisins can be physically trapped in the structure of macromolecules by means of supramolecular interactions. Various extraction solvents used during the isolation of toxins from the matrix can differ in terms of the ability to carry out the general extraction of fumonisins from the matrix. Therefore, despite the fact that there are validated methods that utilise various extraction procedures, an analysis can produce different toxin content results. There is an indirect assay method, which consists of calculating the difference between total fumonisin content after hydrolysis in a strongly alkaline environment and the “free” compounds measured using a “traditional” method [48,49]. Recently, it has been demonstrated that ZEN can also create supramolecular interactions with zein found in maize. The affinity between ZEN and zein depended on the environmental conditions. An acidic environment facilitated the production of “hidden” ZEN, while an alkaline environment caused the toxin to be released [50,51,52]. Selected “modified” toxins produced by the *Fusarium* fungi, which may contaminate maize kernels, are presented in Table 2.

In recent years, particular attention has been paid to so-called “emerging mycotoxins”. This informal term refers to mycotoxins that were identified in the past and are not routinely assayed or covered by standards; however, understanding their biosynthesis, toxicological properties, and their natural occurrence in food has become increasingly important. This group consists of a broad range of secondary metabolites of *Fusarium* fungi, which vary in terms of their chemical structure [26]. It includes moniliformin (MON), BEA, ENNs, and FUS-X; which often co-occur in maize kernels with free mycotoxins [53].

Their simultaneous presence in maize kernels may be associated with the growth of *F. temperatum* and *F. subglutinans* on the plants [14,54]. MON is a sodium or potassium salt of 1-hydroxycyclobut-1-ene-3,4-dione, isolated by Cole et al. [55] from maize kernels infected by *F. verticilioides*. BEA and ENNs belong to closely related groups of cyclodepsipeptides, produced by numerous Fusarium species (most often *F. avenaceum* and *F. poae*), which can have insecticidal, antibiotic, and cytotoxic properties [55]. ENNs is a large group of compounds, consisting of approximately 26 natural analogues [56]. FUS-X is a bicyclic sesterterpene, produced primarily by *F. proliferatum*, *F. subglutinans*, and *F. temperatum* [54]. As the research into mycotoxins progresses (which is firmly linked to the access to commercial references substances), new chemical compounds continue to join the group of candidates for “emerging” mycotoxins. They may include certain trichothecenes (e.g., MAS, DAS, NEO); culmorin (CUL) and its derivatives; as well as other substances, whose presence in maize has been confirmed by various authors [26,36,37]. Selected “emerging” mycotoxins, characteristic of the *Fusarium* species which infect maize, are presented in Table 3.

## 3. Occurrence

### 3.1. Free and Modified Mycotoxins

Literature data which summarises the incidence of mycotoxins and their modified forms, along with their concentrations in maize kernels, are presented in Table 4. The mycotoxins that were the most often identified in maize kernels were fumonisins, DON, ZEN, and—to a lesser degree—their modified derivatives, which co-occurred with native toxins, generally at significant concentrations. They can increase the toxicity of maize which reaches the consumers in the form of maize products, as well as constitutes a basic raw material for the manufacture of animal feed. The increase in general toxicity may occur directly or indirectly by way of transformation to the native form during digestion in the gastrointestinal system [26].

In accordance with literature data published in recent years, the incidence of fumonisins in maize was reported as high [26,57,58,59,60,62]. The presence of FB_1_ in maize kernels, which depended on the origin of the plants, was found in nearly 34% of samples (Table 4). The highest concentrations of FB_1_ were found in material originating from Michigan (USA) (45,145.82 µg/kg) [60]. The highest concentrations of FB_2_ and FB_3_ (22,538.63 and 17,972.72 µg/kg, respectively) were also recorded from this location [60]. In samples from other locations, fumonisin content was lower. Ekwomadu et al. [26] found significant fumonisin contamination (8908, 3383, 990, 1014 µg/kg for FB_1_, FB_2_, FB_3_, and FB_4_, respectively) in various maize varieties originating from two agricultural regions of South Africa [26]. Furthermore, Oliveira et al. [65] tested 57 samples of maize from Brazil for mycotoxins and all of them contained FB_1_ and FB_2_, with a maximum total toxin content of 66,274 µg/kg [65]. A high level of fumonisin contamination in maize is firmly linked to its growing conditions—the warmer and more humid the climate, the greater the likelihood of mycotoxin presence in the kernels. In the context of modified forms of fumonisins, there is little literature on their incidence in maize kernels. Some authors demonstrate that the majority of fumonisins can occur in maize kernels in modified form. Hu et al. [58] confirmed this hypothesis in two types of samples (raw maize kernels and maize products) [58]. That study demonstrated the presence of free fumonisins (FB_1_ + FB_2_) in 66% of all examined samples from both groups; and this increased to 86% following the alkaline hydrolysis of the samples (total fumonisin content after hydrolysis). In the case of fresh maize kernels, 75% of samples tested positive (FB_1_—min–max < LOQ–49 µg/kg); after taking into account free and modified FB_1_, those values ranged between 78 and 131 µg/kg. FB_2_ was not found in those samples, either during direct analysis or after hydrolysis. A similar trend was observed in maize kernel samples infected with a highly toxicogenic pathogen. The maximum FB_1_ and FB_2_ content in maize kernels was 24,890 and 5506 µg/kg, respectively, and 49,782 and 8417 µg/kg after alkaline hydrolysis of the samples [58]. The presence of modified fumonisins in maize can pose a significant problem in terms of real exposure of humans and animals to this group of compounds; however, there is very little data on the presence of total fumonisins (in free and modified form) in maize kernels. The presence of hidden, hydrolysed and partially hydrolysed fumonisins in naturally contaminated maize kernels was also confirmed in our earlier studies [71].

ZEN is a toxin which is also frequently identified in maize; its presence is determined by the location of the growing area. The maximum content of that toxin in raw maize kernels ranged between 79 and 4148.75 µg/kg [26,57,58,59,60,62,65,66], whereas the maximum content of ZEN in maize kernels was recorded in Michigan (similarly to fumonisins) [60]. The presence of ZEN in maize samples is mainly associated with the development of GER disease in maize plants. Usually, the disease is caused by *F. graminearum*, but also other related species such as, for example, *F. culmorum*. The presence of these two species may also be determined by the geographic location of the crop. GER is dominant in areas with moderate temperatures and a relatively higher level of rainfall during the growing season. Other factors influencing the spread of pathogens, and thus increasing the likelihood of contamination of the ZEN maize grain, are plant damage caused by insects and the storage of maize grain with inadequate humidity [36]. Very few scientific publications contain data on the incidence of modified forms of ZEN, i.e., ZEN-14S, ZEN-14G, α- and β-ZOL. The relationships between the occurrence of individual compounds in the maize mill are also not known. Their incidence and concentration levels are usually lower than in the basic analogues. Birr et al. [66] identified α-ZEL in 59% of maize kernel samples (maximum content was 423 µg/kg) and its content depended on the location of the growing areas in Europe [66]. β-ZEL was less common (32% of samples); its maximum content was also lower (203 µg/kg). As for ZEN-14G and ZEN-14S, their maximum concentrations were 274 and 51 µg/kg, respectively [46]. In recent years, “hidden” ZEN forms were also found, similar to the case of fumonisins. Their presence likely results from the supramolecular interactions between zein found in maize kernels and ZEN, and in some cases, it may exceed the content of the basic analogue [52]. Tan et al. [72] found that ZEN in maize kernel samples ranged from <LOD to 163.58 µg/kg, while the hidden form content (calculated as the difference between the total ZEN content after hydrolysis and the free ZEN content) ranged between 0 and 54.53 µg/kg [72].

The presence of DON in maize grain is related, as in the case of ZEN, to the presence of GER on maize plants. Harvest residues such as maize stalks and wheat straw are the main source of *F. graminearum* and *F. culmorum*. Furthermore, the optimal conditions for water activity and temperature for the development of these species differ, so that *F. graminearum* may dominate in one location and *F. culmorum* in the other. These two species are able to biosynthesize not only DON, but other type B trichothecenes (NIV, FUS-X, 3-AcDON and 15Ac-DON). Both DON and NIV chomotypes are identified within *F. graminearum* and *F. culmorum*. *F. cerealis* is a related species and is considered only a producer of NIV [22,33,36]. However, DON was found most often and in the highest content in maize grain. Its biosynthesis is facilitated in cooler regions of Europe and North America, but some authors suggest that this toxin can also occur in maize kernels originating from warmer growing areas [26]. The DON content in maize was strongly correlated with the content of the DON metabolite (DON-3G) [66]. The maximum content of DON in maize kernels ranged between 83 and 20,475 µg/kg [26,57,59,60,63,65,66,67,68] with the highest content in maize from Michigan (20,475 µg/kg) and Germany (10,975 µg/kg) [60,66]. In maize samples originating from regions of South Africa, the maximum reported level of DON was 1380 µg/kg [26]. Among all modified forms of DON, the majority of data is for DON-3G. The highest content of DON-3G, similar to DON, was found in maize samples from Michigan (max. 6266.49 µg/kg) [60]. Birr et al. [66] also confirmed the presence of DON-3G in all their samples. Its maximum content was 3038 µg/kg, which represented 28% of the maximum content of DON [66]. The content of 3-AcDON in maize kernels ranged between 63.04 and 1046.8 µg/kg [46,57,60,70], with the highest concentration detected in maize from Belgium [70]. In the case of 15-AcDON, another DON derivative, the highest content in maize kernels (1787.6 µg/kg) was from Michigan [60]. At 35.7–142 µg/kg, NIV in maize kernels was relatively low compared to DON [26,57,64].

T-2 and HT-2 toxins were significantly less common in maize kernels compared to other toxins produced by *Fusarium* (Table 4). Natural occurrence of T-2 toxin is mainly connected with the tropical and subtropical regions. T-2 toxin can be metabolized into HT-2 toxin. The most important factors that influence T-2 toxin production are weather conditions, grain defects and moisture content (13–22%) [28,36]. T-2 toxin is produced at a wide temperature range (0–32 °C), with maximum production at temperatures below 15 °C. For example, *F. sporotrichioides* has a low optimal temperature (6–12 °C) for T2 toxin production and can produce this mycotoxin during overwintering in the field and/or during storage. Among all grains, corn, wheat, barley, oat, and rye are most frequently contaminated with T2-toxin [46]. T-2 toxin was found in maize samples from Michigan in no more than 19% of the examined samples, at concentrations of <LOD–156.65 µg/kg. HT-2 was also rarely found in maize samples (in no more than 5% of samples) and the maximum content ranged between 40.2 and 276.74 µg/kg, depending on the sample origin [26,60].

### 3.2. Emerging Mycotoxins

There is limited literature data on the occurrence of “emerging” mycotoxins. However, in recent years there has been an increased interest in this topic. These compounds can be found all over the world where maize is grown and they can coexist with toxins with free as well as modified mycotoxins. The literature data indicate that some of the “emerging” mycotoxins can occur at significant concentrations (e.g., MON, BEA and FUS). Those toxins were found in nearly all maize samples. Emerging mycotoxins naturally occurring in maize kernels are presented in Table 5.

MON was found in the majority of maize samples at relatively high concentrations as compared to other “emerging” mycotoxins. The maximum content of that toxin in maize kernels ranged between 116 and 4800 µg/kg [26,54,57,60,64,68]. Relatively high MON content was recorded in Serbia, where the maximum values ranged between 850 and 3856 µg/kg, depending on the growing season [54]. In Italy, MON concentration ranged between 751 and 4800 µg/kg, also depending on the growing area [68]. On the other hand, MON was found in as many as 98% of South African samples at a maximum concentration of 1130 µg/kg [26]. A similar incidence (87%) and a high content in maize kernels were recorded for BEA. As shown in Table 5, the maximum concentration of BEA ranged between 18.2 and 7446.21 µg/kg [26,54,57,60,64], whereas the most contaminated kernels originated in Michigan (max. 7446.21 µg/kg) [60]. Conversely, all maize samples originating from Nigeria were confirmed to contain BEA at a relatively lower maximum concentration, 329 µg/kg [64]. ENNs are a group of compounds which are structurally similar to BEA but occur in maize kernels less often. Among ENNs, ENN B was the most common (27% of samples) and occurred at the highest concentrations. Its maximum content was found in maize kernels from Belgium (1984.9 µg/kg) [70]. There has been little data published in recent years concerning the incidence of FUS in maize kernels [54,64]. Research carried out in Serbia confirmed a high incidence of FUS at an average of 64.1% across all examined regions; the highest FUS concentration ranged between 1121 and 12,272 µg/kg [54]. In Nigerian maize kernel samples, both the incidence and content of FUS were relatively low [64]. Other toxins, such as MAS, DAS or NEO, were much rarer and their concentrations were relatively low (Table 4).

There is very little literature data concerning other “emerging” mycotoxins, such as CUL, FA, fusarinolic acid, aurofusarin, 15-hydroxyculmorin and 5-hydroxyculmorin. Ekwomadu et al. [26] suggested that the content of these compounds may be significant, while Reisinger et al. [63] suggest that their concentration may be higher in maize silage [26,36]. However, more extensive research is required to more precisely estimate the concentration of those compounds in maize kernels.

## 4. Toxicology

### 4.1. Free and Modified Mycotoxins

#### 4.1.1. Trichothecenes and Their Modified Forms

The toxicity of type B trichothecenes results from their ability to bind to ribosome subunits, thus inhibiting protein synthesis. This process is referred to as ribotoxic stress [73,74]. The EFSA Panel on Contaminants in the Food Chain has set the TDI value (Tolerable Daily Intake) for the sum of DON, 3Ac-DON, 15Ac-DON and DON-3G at 1.0 μg/kg of body weight [75]. In recent years, multiple studies evaluated the cytotoxicity of DON and its metabolites. In those studies, cell lines representing the gastrointestinal organs were used, including: HepG2 (liver), Caco-2 (small intestine), IPEC-J2 (porcine small intestine), and GES1 (stomach). The only metabolite of DON whose toxicity was comparable to its native form was 15-acetyldeoxynivalenol (15-AcDON) [75,76,77,78]. On the other hand, metabolites such as 3-AcDON, DON-3G, and deepoxy-deoxynivalenol (DOM-1) were characterised by significantly lower toxicity [76,77,79]. The acetylated derivatives of DON are linked with the ribosome by two hydrogen bonds and the native form of DON by three hydrogen bonds. The acetylated groups in DON metabolites influence the strength of the bond in the toxin-ribosome complex. The acetyl group at C-3 induces stabilising van der Waals forces, while the same group at C-15 causes the formation of an additional, stabilising hydrophobic interaction [80,81]. It is suggested that esterification at C-15 increases toxicity and acetylation at C-3 causes it to decrease [78]. In recent years, there have been reports that DON and its acetylated metabolites may induce oxidative stress in the intracellular environment. Following the exposure of GES-1 cells to 15-AcDON and DON, they were observed to have a higher concentration of reactive oxygen species, disturbed NAD+/NADH balance, and reduced ATP level [77]. Furthermore, the identification of malondialdehyde (MDA), a marker of lipid peroxidation in cells exposed to those compounds, suggests that they can induce that process [82]. The induction of oxidative stress in cells results in DNA damage, which in turn causes the formation of neoplastic lesions. Studies on cell lines demonstrated that exposure to DON and 15-AcDON caused DNA damage, which disturbed the cell cycle, and enhanced the expression of genes responsible for the antioxidant protection [78,82]. DON and 15-AcDON have proapoptotic effects [77]. The most recent studies on the GES-1 cell line and porcine hippocampus cells found that this process also involves mitogen-activated protein kinases (MAPK) p38 and JNK, as well as ERK1/2 kinases [77,83]. NIV’s structure is nearly identical to DON’s; therefore, both substances have similar toxic effects. Both compounds inhibit cell proliferation, induce the production of IL-8, activate kinases from the MAPK family and engage the κB nuclear factor in the toxicity signal transduction pathways [84]. In the majority of studies that compared NIV’s and DON’s toxicity in in vitro tests on cell lines found significantly higher cytotoxicity of NIV as compared to DON. In studies on cell lines of promyelocytic leukaemia (HL60), lymphoblastic leukaemia (MOLT-4) and rat aorta myoblasts (A-10), IC50 values (concentration which inhibits cell proliferation by 50%) for NIV were several times lower than those for DON [85,86,87,88]. However, in HepG2 cells, IC50 values for both compounds were comparable [84]. As opposed to DON, NIV did not significantly induce the secretion of anti-hematopoietic cytokines, CCL3/CCL4, in the cells [84]. Therefore, it can be assumed that for leukopenia caused by type B trichothecenes, in the case of NIV, it is the result of cytotoxicity towards leukocytes, and in the case of DON it is the result of both cytotoxicity and the inhibition of leukocyte production. The few publications describing the toxicity of NIV metabolites, usually only include fusarenone X (FUS-X) and found that this compound has similar or slightly lower toxicity compared to its native form. These observations were confirmed in GES-1 cell lines and human T cells (Jurkat Cells) [77,89]. Mouse studies demonstrated significantly greater DNA fragmentation in the thymus gland, Peyer’s patches and spleen in animals exposed to FUS-X than in those exposed to NIV [90]. It is likely that nivalenol-3-glucoside (NIV-3G) is not effectively hydrolysed to NIV in an in vivo environment, as observed in rat studies. The analysis of urine of animals exposed (using a tube) to NIV-3G showed a 30-times lower content of NIV compared to the group of animals who received equimolar doses of NIV [91].

Type A trichothecenes exhibit significantly greater toxicity than other types of trichothecenes. Their toxicity mechanism is based on binding with a large ribosome subunit and inhibiting translation [92]. Those compounds exhibit neurotoxicity, hepatotoxicity, hematotoxicity, and reproduction toxicity. Acute poisoning causes nausea, vomiting, diarrhoea, damage to the stomach and liver, and weakened immunity [93,94]. The EFSA Panel on Contaminants in the Food Chain determined the TDI value for the sum of T-2 and HT-2 toxins at 0.02 μg/kg of body weight [95]. Numerous in vitro studies on cell lines representing liver, intestinal and kidney cells, murine macrophages, and porcine Leydig cells demonstrated comparable toxicity of T-2 and HT-2 toxins [96,97,98]. The similar toxicity of those compounds may be the result of the effective transformation of the T-2 toxin into the HT-2 toxin in the intracellular environment. Studies on the HepG2 line showed that the effectiveness of biotransformation of the T-2 toxin to the HT-2 toxin reached 94% [96]. Toxicity similar to that of the T-2 toxin can be also found in DAS. This compound can decrease the viability of intestinal cells, liver cells, and murine macrophages to a similar degree [99].

In recent years, significant attention has been paid to the induction of anorexia by type A trichothecenes. This process involves intestinal satiety hormones, cholecystokinin (CCK) and glucagon-like peptide 1 (GLP-1), as well as neurotransmitters—substance P (SP) and serotonin, also referred to as 5-hydroxytryptamine (5-HT) [100,101]. In mink, the induction of neuropeptide YY (PYY) production occurred as early as 30 min following oral or intraperitoneal administration of DAS (a type of trichothecene) [102]. Unfortunately, the impact of the T-2/HT-2 toxins on PYY synthesis has not yet been described and there is no information on the toxicity of T-2/HT-2 glucosides in the literature. It is suggested that those compounds may be characterised by effective intestinal absorption; Broekaert et al. [103] reported that T-2 toxin-α-glucoside in the gastrointestinal tract of broiler chickens was absorbed five times faster than its aglycone [103].

#### 4.1.2. ZEN and Its Modified Forms

The toxicity of ZEN results from its ability to cause oxidative stress, damage DNA, inhibit the cell cycle and induce apoptosis [101,104,105,106]. The EFSA Panel on Contaminants in the Food Chain determined the TDI value for ZEN at 0.25 μg/kg of body weight [107]. In vitro tests showed greater cytotoxicity of the hydroxylated ZEN metabolite, i.e., β-ZOL, as compared to the native toxin. The cytotoxicity of α-ZOL compared to ZEN is controversial—depending on the cell lines used, studies produce different results [99,108,109]. Furthermore, the data on the cytotoxicity of ZEN-14G is limited. At a concentration of 1 μM, this compound did not have a significant effect on the viability of MCF7 cells (breast cancer cells) [110]. Similarly, its cytotoxicity on small intestinal cells (Caco-2) at concentrations of 20 and 40 μM has not been demonstrated [111]. In accordance with the current knowledge, the cytotoxicity of zearalenone-14-sulfate (ZEN-14S) is unknown. In the case of ZEN and its hydroxylated metabolites (α- and β-ZOL), immunosuppressive effects have been described. Those compounds significantly inhibited the expression of pro-inflammatory cytokines, such as IL-1β, IL-8, and TNF-α [112]. In vitro tests on mononuclear porcine peripheral blood cells exposed to ZEN, α-ZOL, and β-ZOL demonstrated a decrease in neutrophil viability and inhibition of IgG antibody production [99,110]. Furthermore, α-ZOL inhibited the proliferation of T cells [113]. Unfortunately, α-ZOL and ZEN were not tested for this ability; thus, it is not known whether they have similar properties.

An important aspect of ZEN’s toxicity is its xenoestrogenic activity. With a structure similar to oestrogen, it can stimulate oestrogen receptors and disturb the metabolism of steroids and the proliferation of oestrogen-dependent cells [114,115,116]. Long-term exposure to xenoestrogens can cause numerous medical conditions, including disrupted puberty, obesity, infertility (in males), and cancers (breast, ovarian, testicular and prostate) [30,117]. In recent years, researchers have described the xenoestrogenic activity of hydroxylated ZEN metabolites. Significantly, α-ZOL was identified as a stronger xenoestrogen than ZEN [118,119]. Additionally, the affinity between ZEN-14G and oestrogen receptors is several hundred times lower than that of ZEN. However, in the cellular environment this compound is hydrolysed to xenoestrogens, such as α- and β-ZOL [110,120]. The activation of oestrogen receptors can alter gene expression, since those receptors influence the activity of numerous transcription factors [118,121]. It has been reported that α-ZOL and ZEN produce significant epigenetic changes, and cause DNA methylation and acetylation of histones in the HepG2 cell line. These changes impair the expression of many crucial metabolism genes, including the genes that regulate glucose and lipid metabolism, and those that regulate insulin secretion. It is suggested that these changes may contribute to the development of type 2 diabetes [108].

#### 4.1.3. Fumonisins and Their Modified Forms

The main aspect of fumonisin toxicity is the inhibition of ceramide synthesis and the disruption of sphingolipid metabolism, which can cause cell apoptosis [122]. This mechanism of action stems from the similarity between the chemical structure of fumonisins and the structure of sphingosine (the primary part of sphingolipids), which is why they are recognised, and bound, by ceramide synthase [123]. The EFSA Panel on Contaminants in the Food Chain determined the TDI value for the sum of FB_1_, FB_2_, FB_3_, FB_4_ at 1.0 μg/kg of body weight [124]. The likely teratogenic activity of FB_1_ is by intercalating with the folic acid transporter, and thus limiting the possibility of the folic acid being captured by the embryo. Due to the crucial role of folic acid in the development of the nervous system in embryos, FB_1_ exposure may cause foetal neural tube defects [125]. There are numerous publications which demonstrate that fumonisins induce oxidative stress. Along with the disturbance of sphingolipid metabolism, this process is the primary mechanism of FB_1_ toxicity, which can lead to lipid peroxidation and DNA damage [126,127,128,129]. When exposed to FB_1_, rats suffer DNA damage, the extent of which depends on the dose [130]. The genotoxicity of FB_1_ in in vitro testing is not clear. Following exposure to FB_1_ no DNA damage was observed in human kidney and liver cells [131]. However, recent studies on human oesophageal epithelial cells suggest that this compound may promote carcinogenesis. FB_1_ significantly limited the expression of suppressor genes that inhibit the cell cycle in response to DNA damage [132].

The exposure of various animal species to fumonisins produced different symptoms: horses and mice suffered damage to the nervous system; in ruminants, the characteristic symptoms included hepatotoxicity, reproduction toxicity and weakened immunity [133]. In rodents, FB_1_ exposure caused liver damage, which could lead to liver cancer [134]. Existing toxicological studies suggest that this toxin can cause oesophageal cancer and bleeding, chronic cough, indigestion and weight loss in humans [125]. As a result of 48-h exposure of GES1 line cells (gastric epithelium) to 40 μM of FB_1_, FB_2_, and FB_3_, the following toxicity hierarchy was established: FB_1_ > FB_2_ > FB_3_ with cell mortality rates of 48.44%, 34.66%, and 27.37% respectively. Furthermore, a synergistic effect was found during the exposure to a mixture of fumonisins, i.e., FB_1_/FB_2_ and FB_1_/FB_3_, and the primary type of cell death was necrosis [135].

There are a very limited number of publications describing the toxicity of modified forms of fumonisins. In in vitro tests, the toxicity of HFB_1_ and HFB_2_, as compared to FB_1_, ranged between 0.01 and 0.9. Significantly, the absorption of HFB_1_ by the cells was more effective than FB_1_ [124]. Recently, it has been reported that HFB_1_ is less able to induce the expression of pro-inflammatory chemokines IL-8 and CCL20, as well as cytokine TNF-α in the IPECJ2 (porcine small intestinal cells) and PBMC (mononuclear cells of peripheral blood) cell lines [136].

### 4.2. Emerging Mycotoxins

Owing to the numerous reports made in recent years, the issue of toxicity of compounds classified as “emerging” mycotoxins has been increasingly common. At the same time, the multitude of compounds in that group, as well as the fact that new mycotoxins are regularly identified, means that the amount of available information concerning this topic is insufficient. Furthermore, because of the limited data on the toxicity of “emerging” mycotoxins, as well as their incidence in foodstuffs, it is impossible to suggest their permissible content in cereals. Therefore, there is currently no legislation that would regulate the content of compounds from this group in food [137,138,139].

The mechanism of toxicity of ENNs and BEA is likely related to their ionophoric properties. By penetrating cellular membranes, those compounds create cation-selective channels, causing ionic imbalance in the cells. Elevated levels of calcium ions in the cells activate caspase-3 through the release of cytochrome C and lead to apoptosis [137,140,141]. ENNs and BEA also have cytotoxic effects as a result of the induction of oxidative stress [142,143]. Following oral exposure of rats to a mixture of enniatin’s A, A1, B and B1, a change in the expression of genes responsible for the antioxidative protection, genes of suppressor proteins, as well as electron carriers (NADPH) was observed in their stomach, kidney, liver and intestinal cells. Furthermore, the abnormal activity of occludin (a tight junction protein) in the intestines suggests that these compounds may impact the permeability of the intestinal barrier [143].

Another relatively well-studied *Fusarium* toxin classified as an “emerging” mycotoxin is MON. Its mechanism of action is based on an inhibition of pyruvate dehydrogenase and α-ketoglutarate dehydrogenase, thus disturbing the Krebs cycle and causing ATP deficiency. The cytotoxicity of MON in in vitro tests largely depends on the cell lines used. Although not cytotoxic towards human progenitor cells of leukocytes and platelets, it significantly reduces the viability of progenitor cells of erythrocytes. In livestock, MON exposure caused muscle weakness, heart failure, and respiratory failure [144]. Data concerning FUS is much more limited. Based on a study on *Artemia salina*, the LD50 value for this compound was determined at 53.4 µM. Comparable LD50 values were obtained following the exposure of *Artemia salina* to DON, suggesting that the toxicity of those compounds is similar. Fusaproliferin may have teratogenic properties, as demonstrated in the studies on hens. Macrocephaly, cephalic dichotomy and leg deformations were observed in embryos injected with fusaproliferin [34]. In vitro tests using human small intestinal cells demonstrated that the cytotoxicity of the recently identified NX toxin, classified as a trichothecene, was similar to that of DON. A slightly smaller decrease in cell viability was recorded following exposure to 3-acetyl-NX (17.4%), as compared to 21.4% and 20.2% for NX-2 and DON, respectively. The observation of porcine jejunal explants following 4-h exposure to 10 μM of NX-2 demonstrated the presence of intraparenchymal oedema, cell vacuolation, ruptured epithelial barrier, and significant loss of intestinal villi [145]. Interesting observations have been made recently of CUL which although characterised by negligible cytotoxicity, can reduce the effectiveness of glucuronidation of DON by liver cells. This process is the main pathway of DON detoxification in humans; therefore, CUL and DON are likely characterised by synergistic toxicity [146]. Recently, there have been reports of the possible induction of oxidative stress by aurofusarin and fusaric acid [147,148]. However, the available literature data concerning this topic is limited, and further research should be conducted.

The vast majority of studies into the toxicity of secondary metabolites of *Fusarium* fungi take into account only the native toxins. Significantly less attention is paid to the metabolites of toxins which co-occur with the native forms in the environment and can be characterised by comparable toxicity (e.g., 15-AcDON, α-ZOL). Many plant metabolites of toxins produced by *Fusarium* fungi discovered in recent years have not undergone a cytotoxicity evaluation (DON-14S, T-2-Glc, HT-2-Glc). The issue of mycotoxin interactions, which may occur in the in vivo environment and cause antagonistic, additive or synergistic effects, is relatively rarely discussed in the literature. The toxicity evaluation based on in vitro experiments should take into account the properties of the examined compounds in the in vivo environment. Important factors include the potential degradation of the gastrointestinal tract, intestinal absorption, rate of elimination, volume of distribution, and maximum concentration.

## 5. Progress in Detoxification

Currently, the reduction of mycotoxin contamination in various agricultural commodities is a major issue in many nations [149] as they are heat stable compounds. To avoid these concerns, several preventive measures have been implemented, including pre-harvesting procedures aimed at inhibiting the growth of toxigenic fungus and mycotoxins production, as well as post-harvesting strategies aimed at detoxifying food once mycotoxins have been synthesized. Unfortunately, none of these measures guarantee the complete absence of mycotoxins in food or feed. However, it is critical to keep the prevalence of mycotoxins below the threshold of economic impact, which is why so many studies aim to tackle these challenges. Different approaches for inhibiting fungal growth and minimizing mycotoxin production in maize have been explored including prevention and decontamination mechanisms. Fungal growth and mycotoxin prevention includes strategies related to good agricultural practices (GAPs), good manufacturing practices (GMPs), and good hygienic practices (GHPs), while decontamination mostly include chemical, physical and biological techniques [150]. Agricultural practices can include crop rotation, tillage, use of chemicals as well as breaking the fungal disease cycle by adapting the sowing period or using resistant hosts; physical techniques (cleaning, sorting, irradiation, thermal or ultrasound treatment, temperature and humidity control); chemical approaches (acids and bases such as ammonia, hydrogen peroxide, or antifungal agents); and biological control methods made of the use of microorganisms (bacterial, yeast and fungi) and plant products (plant essential oils and plant extracts etc.) [151].

Although these approaches can reduce fungal infection and/or mycotoxins biosynthesis, there are various limitations, problems, and concerns with regards to physical and chemical detoxification. Physical approaches typically lack specificity and efficacy, whereas chemical approaches sometimes necessitate either specialized equipment and expensive chemicals or extreme treatment conditions, which may have an unacceptable negative impact on the environment and non-targeted organisms. Even more significantly, they may pose health risks to humans and animals, and some methods are commonly regarded as impossible, costly, ineffective, laborious, or time-consuming [151,152]. In addition, fungicide-based approaches represent a possible health, safety, and environmental danger because many antifungal chemical compounds are not biodegradable or have a long degradation time, can contaminate soil and water, and have a negative impact on food quality and human health. Prolonged chemical treatment of grains can also result in fungal strains developing resistance, necessitating higher concentrations, and an increase in hazardous residues in food crops [153]. However, driven by health and environmental concerns, various laws addressing the usage of chemical control measures are being established as consumers become more aware of the potential dangers of chemicals in the food supply [154], and there is a societal and ecological drive movement toward safe and natural food that is free of chemical treatments and/or preservatives. Thus, biological approaches have gained more attention due to their benefits such as target specificity, being economically feasible, and being environmentally benign [155]. In this section, a comprehensive overview of the recent biological detoxification of maize is reviewed.

### 5.1. Biological Detoxification of Maize

Currently, different pre-harvest and post-harvest biological control systems have been developed for maize against *Fusarium* spp. and their respective mycotoxins. These have used a variety of potential Biological Control Agents (BCAs), including fungal and bacterial strains or toxigenic fungal strains, botanicals (essential oils, crude plant extracts or phenolic acids). However, most of the reported BCAs are still limited to the in vitro lab scale and are not typically commercialized, and this field is still in its infancy [156]. Many aspects must still be investigated to enable effective integration, and understanding the processes that govern the interaction of BCAs and pathogens throughout time is especially important. Four common modes of actions of BCAs that have been identified and described by various researchers: antibiosis, competition for niche or nutrients, mycoparasitism, and stimulation or enhancement of plant defence [157,158,159]. BCAs typically rely on more than one mode of action to combat the pathogen, and the existence of one dominant mode of action does not preclude the presence of the others [157] and modes may depend on the parameters and kind of BCAs under consideration.

Although many BCAs have been developed and their successful application to fight against mycotoxigenic fungi have been reported, the following paragraphs will only discuss a selection because of their importance in sustainability protection of maize against *Fusarium* spp.

#### 5.1.1. Trichoderma as Biological Detoxifying Organisms

*Trichoderma* are soil-dwelling, free-living filamentous fungi that include rhizosphere-competent strains connected with root ecosystems. As potential antagonistic microbes, the genus *Trichoderma* has been widely studied for their capabilities against plant pathogenic fungi, and their biological control mechanisms mainly include faster growth speed and antibiotic production to compete for nutrients and living space with pathogens, mycoparasitism mediated by producing cell wall degrading enzymes, and the ability to induce plant’s defence systems [157,159,160]. The general mechanisms of *Trichoderma* spp. biocontrol can be separated into direct and indirect impacts. Competition for nutrients or space, synthesis of volatile and non-volatile antibiotics and lytic enzymes, inactivation of pathogen enzymes, and parasitism are all direct consequences. Indirect impacts include morphological and metabolic changes in the host plants, such as stress tolerance, inorganic nutrient solubilization or sequestration, and induction of fungal phytopathogen-caused disease resistance [161].

*Trichoderma* species are efficient bio-controllers of phytopathogenic *Fusarium* [158,162] with the ability to combine numerous benefits in one product, including the control of various plant diseases, the stimulation of plant growth, and the creation of a clean environment for the benefit of sustainable agriculture [157]. In a screening experiment, the abilities of twenty-four isolates belonging to ten different *Trichoderma* species were tested against the mycelial growth and mycotoxin production by five *Fusarium* strains [158]. All the selected strains were capable of affecting the mycelial growth of at least four of all five *Fusarium* species on the fourth day after co-inoculation, when there was the first apparent physical contact between antagonist and pathogen. However, *T. atroviride* AN240 was found to be the most efficient (69–100% toxin reduction) suppressor of mycotoxin (DON, 3-AcDON, 15-AcDON, NIV, ZEN, BEA, MON) production by all five *Fusarium* species (*F. avenaceum*, *F. cerealis*, *F. culmorum*, *F. graminearum* and *F. temperatum*) on solid substrates [158]. Recently, there has been increased interest in managing DON production with *Trichoderma* strains as a biological control-based technique. Eight selected *Trichoderma* strains effectively inhibited *F. graminearum* mycelial growth and mycotoxin DON synthesis. Furthermore, the modified mycotoxin deoxynivalenol-3-glucoside (DON-3G), which was once regarded as a detoxification product of DON in plant defense, was detected when *Trichoderma* interacted with *F. graminearum* [163].

Further, the inhibitory effects of a biocontrol agent based on the *Trichoderma asperellum* isolate GDFS1009 on the management of stalk rot in maize caused by *F. graminearum*, as well as the effect on the maize plant growth were reported with a 60% inhibition rate against *F. graminearum* [164]. Another research group explored the effect of cellulase from *T. harzianum* Th22 on triggering the biosynthesis of 2,4-Dihydroxy-7-methoxy-2H-1,4-benzoxazin-3(4H)-one (DIMBOA) and defence-related gene expressions in maize roots against DON producing *F. graminearum* and it was found that DIMBOA inhibited the pathogenicity and mycotoxin related proteins in pathogen *F. graminearum* [165]. The efficacy of seven *T. asperellum* strains obtained from fields in Southern China was evaluated against *F. graminearum*. The *T. asperellum* ZJSX5003 strain enhanced the antagonist activity against *F. graminearum* and reduced disease prevalence by 71% in inoculated maize plants compared to negative control [166].

Other mycotoxins are also reduced by *Trichoderma* strains. Tian et al. [167] discovered three *Trichoderma* isolates that could successfully limit the mycelia spread and mycotoxin synthesis of ZEN-producing *F. graminearum* [167]. Furthermore, ZEN-treated experiments revealed that although these *Trichoderma* isolates could not detoxify ZEN by glycosylation, they could convert ZEN to its reduced (α-ZOL and β-ZOL) and sulfated metabolites (ZEN14S and ZOL14S), providing more detail on how *Trichoderma* isolates and ZEN-producing fungus interact. In addition, *Trichoderma harzianum* strains have received special attention. In controlled and uncontrolled environments, seeding maize with the *T. harzianum* T22 and Th-8 strains lowered *F. verticillioides* kernel colonization and FBs contamination, as well as generated systemic resistance in maize against these pathogens [158]; also *T. harzianum* T16 and T23 strains have been found to be effective antagonists towards *F. verticillioides* and FBs production in maize kernels in liquid as well as agar medium.

#### 5.1.2. Lactic Acid Bacteria as Biological Detoxifying Agent

Lactic acid bacteria (LAB) are a group of related bacteria (e.g., *Streptococcus* spp., *Lactobacillus* spp., *Lactococcus* spp., and *Leuconostoc* spp.) that produce lactic acid as a result of carbohydrate fermentation [168]. LAB occur naturally in foods and are either considered to be harmless or possibly supportive of human health in their capacity as probiotics. LAB is one of the most studied microorganisms for mycotoxin degradation due to their high safety profile in food applications. They produce various bioactive compounds such as organic acids (acetic and lactic acid), hydrogen peroxide, proteinaceous compounds, reuterin, hydroxyl fatty acids, and phenolic compounds that can inhibit fungal development and prevent the generation of mycotoxins in food [169]. The detoxification ability of LAB can be attributed to mycotoxin adsorption by the bacterial cell structure or degradation via its metabolism [170].

The ability of LAB to inhibit fungal development and remove several mycotoxins, as well as its general safety and probiotic potential, make it a promising candidate for the biological control of fungi such as *F. graminearum* or *F. verticillioides* in maize and during food production and processing. The most prevalent bacterial isolates associated with anti-*Fusarium* action are *Lactobacillus* spp. with *L. plantarum* appearing to be particularly efficient [169]. Li et al. [170] investigated the effect of lactic acid bacteria on the fermentation quality and mycotoxin concentrations of corn silage infested with mycotoxigenic fungi [170]. They found all inoculants decreasing the DON and FB_1_ concentrations, while only *L. buchneri* and *L. plantarum* reduced the ZEN. Researchers investigating whether LAB fermentation (using indigenous microflora) reduced FB_1_ and ZEN concentrations in fermented maize meal found a significant decrease in the concentration of the two mycotoxins with a 68 to 75% for ZEN and 56–67% for FB_1_ [171]. Similarly, Franco et al. [172] reported that LAB inhibits *F. graminearum* and DON detoxification [172]. The application of the LAB as detoxifying agent is still limited in maize and maize products intended for human consumption. Although its application in silages and feed maize continues to develop [170,173,174], further research is still needed.

#### 5.1.3. Plant Secondary Metabolites as Biocontrol Detoxifying Agent

Essential oils, spices, herbs, and crude extracts from plants are excellent candidates for the development of bio-fungicides and nutraceuticals to treat mycotoxicosis and associated infections. In general, botanicals are widely recognized as environmentally friendly and safer sources of bioagent for the prevention of fungal growth and mycotoxin biosynthesis in food and feed. They are less expensive than other materials used for the same purpose, provide a synergistic approach as protectants against fungal/mycotoxin contamination, and trigger pathways that elicit natural defence systems in plant tissues.

##### Essential Oils

Essential oils (EOs) are natural compounds extracted from different plant parts including the roots, fruits, flowers, and seeds. They are characterised by their odour that result from the aromatic compounds, terpenes and other substances synthesised by plants as secondary metabolites [175]. The production of EOs and their constituents is influenced by intrinsic and extrinsic factors, including environmental conditions such as climate, rainy and sunny periods, seasonality, and others; and these factors have a direct impact on the majority of compounds, which in turn act on a variety of microorganisms [176]. Essential oils suppress fungi growth and mycotoxin synthesis through a variety of mechanisms, including altered fungal growth rate and lag phase, disruption of cell permeability, disruption of the electron transport chain, and manipulation of gene expression patterns and metabolic processes [177].

Recently, various types of essential oils; such as cinnamon, verbena, palmarosa, orange, and spearmint, *Litsea cubeba*; have been reported to have inhibitory effects on *Fusarium* growth and mycotoxins detoxification [178,179]. Perczak et al. [179] investigated the effects of cinnamon, palmarosa, orange, and spearmint EOs on the growth of *F. graminearum* and *F. culmorum* and the biosynthesis of mycotoxins in maize seeds and found that these EOs have a significant ability to prevent *Fusarium* fungal growth (*F. graminearum* and *F. culmorum*) and in maize seed reduced mycotoxin concentrations of ZEN (99.10–99.92%) and DON (90.69–100%) [179]. However, the efficiency of three cinnamon, verbena, and palmarosa in decreasing ZEN and DON in maize grains is concentration dependant [179]. Additionally, essential oils with certain constituents extracted from aromatic plants (*Aloysia polystachya*, *Origanum vulgane*, *Mentha piperita*, and *Aloysia triphylla*) suppressed the growth of *F. verticillioides* and fumonisin production in maize grain [180]. Castro et al. [175] evaluated the antifungal and anti-mycotoxigenic activity of essential oils (EOs) from *Zingiber officinale*, *Cinnamomum zeylanicum*, and *Cymbopogon martinii* against *F. verticillioides* and fumonisins biosynthesis and found that all tested EOs had inhibitory effects on *F. verticillioides* by promoting structural damage to the fungal cell wall, decreasing conidia size, and mycelial reduction [175]. Similarly, in vitro examination of the antifungal effects of *Litsea cubeba* essential oil against *F. verticillioides* revealed that the investigated EOs significantly decreased FB_1_ and FB_2_ synthesis as well as the mycelial development of *F. verticillioides*. The minimum inhibitory concentration of the EOs was 125 µg/mL and the inhibitory effect was dose dependent [181]. Moreover, multiple essential oils (cedarwood, cinnamon leaf, cinnamon bark, white grapefruit, pink grapefruit, lemon, eucalyptus, palmarosa, mint, thymic, and rosemary) on ZEN reduction under various in vitro conditions, including the influence of temperature, pH, incubation time and mycotoxin and essential oil concentrations have also been explored [182]. In another study, cinnamon oil was effective in reducing FB_1_ from 15.03 to 0.89 μg/mL (94.06%) [183]. Generally, essential oils from plants offer hope in the prevention and detoxification of *Fusarium* mycotoxins present in cereals.

##### Plant Extracts

Plants contain antimutagens, antimicrobials, antioxidants, and anticarcinogens that can mitigate the harmful and genotoxic effects of mycotoxins. Several substances derived from plant extracts have been shown to decrease the growth and toxin generation of *Fusarium* spp. Plant extracts contain a variety of chemicals, including polyphenols, phenolic acids, and flavonoids, which may serve as the biological basis for their antimicrobial activities. Furthermore, plant extracts having antimicrobial capabilities have been utilized to control mycotoxigenic fungus in foods and feeds, potentially eliminating or reducing the use of synthetic chemicals [184]. *Equisetum arvense* and *Stevia rebaudiana* extracts significantly reduced the growth of *F. verticillioides* [185]. Seepe et al. [186], in in vivo experiments of the antifungal effectiveness of various plant extracts against *Fusarium* pathogens on maize seeds, found that Melia azedarach acetone extract had strong antifungal activity (97% inhibition) against *F. proliferatum*, while combined acetone extracts from *Combretum erythrophyllum* and *Quercus acutissima* had 96, 67, and 56% inhibition against *F. verticilloides*, *F. proliferatum*, and *F. solani*, respectively [186]. Furthermore, natural phenolic acids (caffeic, ferulic, p-coumaric, and chlorogenic) inhibit *Fusarium* growth and mycotoxin production in culture medium and in maize kernels [187]. Currently, the application of plant extracts to inhibit *Fusarium* growth and mycotoxins biosynthesis in maize grains is still limited. However, their experimental use gives hope for their application in near future. Recently, the in vitro efficacy of sixteen extracts derived from eight natural sources using subcritical water extraction at two temperatures was assessed against fungal growth and type B trichothecene (TCTB) production by *F. graminearum*. Maritime pine sawdust extract was shown to be exceptionally efficient, leading to a significant inhibition of up to 89% of the fungal growth and a reduction of up to 65% of the mycotoxin production by *F. graminearum* [188]. Furthermore, a study by Uwineza et al. [24] reported the antifungal and mycotoxins reduction of lemon balm extracts against *F. proliferatum* and *F. culmorum* [24].

### 5.2. Edible Mushrooms Source of Biological Detoxifying Agent

Edible mushrooms are macro fungus with a specific fruiting body, which can be either a *Basidiomycete* or an *Ascomycete*, aerial or underground, and large enough to be seen with naked eye and to be collected by hand [189]. Mushroom oyster or white-rot fungus (*Pleurotus ostreatus*) is one of the most well-known edible mushrooms due to its economic (edible), ecological (bioremediation agents), and medicinal value (antioxidant activity and bio-compounds source) [190,191]. Its use as a biocontrol organism for mycotoxin detoxification in cereals especially maize has grown in popularity in recent years [192,193,194] owing to its bioactive compounds (phenolic compounds and proteins) [188,194], and its highly efficient enzymatic systems (manganese peroxide and laccases) for degrading mycotoxins [191,195,196].

Furthermore, other forms of mushrooms have been employed to inhibit *Fusarium* growth and their mycotoxins biosynthesis, since its employment as a natural detoxifying agent has been predicted for the future. Merel et al. [197] studied the in vitro effect of crude extracts (CEs) from *A. subrufescens*, *L. edodes*, and *P. ostreatus* fruiting bodies on the production of biomass and mycotoxins by two strains of *F. verticillioides* mostly contaminating the maize [197].

## 6. Conclusions

Accelerating climate change and the cosmopolitan nature of *Fusarium* fungi significantly affect the profile of these microorganisms present in maize crops, and thus the quality and quantity of mycotoxins. Additionally, the presence of modified forms of toxins and the lack of legal regulations in this regard exacerbates the risk of an increase in their concentration in both raw materials and cereal products. Various biological methods are very promising and safe for the environment and the consumer, and may significantly limit the growth of *Fusarium* and thus the biosynthesis of mycotoxins.

## Figures and Tables

**Table 1:** Important mycotoxins and *Fusarium* species responsible for their biosynthesis in maize kernels.

Mycotoxin	Abbreviation	Molar Mass [g/mol]	Produced by:	Source
Fumonisin B_1_	FB_1_	721.83	*F. dlamini* *F. globosum* *F. nygamai* *F. oxysporum* *F. proliferatum* *F. subglutinans* *F. temperatum* *F. thapsinum* *F. verticillioides*	[26,32,33,34,35,36,37]
Fumonisin B_2_	FB_2_	705.83		
Fumonisin B_3_	FB_3_	705.83		
Fumonisin B_4_	FB_4_	689.83		
Fumonisin A_1_	FA_1_	763.90		
Zearalenone	ZEN	318.36	*F. cerealis* *F. culmorum* *F. equiseti* *F. graminearum* *F. heterosporum* *F. meridionale* *F. semitectum*	[26,32,33,36,37,38]
HT-2 toxin	HT-2	424.48	*F. acuminatum* *F. armeniacum* *F. langsethiae* *F. poae* *F. sporotrichioides*	[26,33,36,37]
T-2 toxin	T-2	466.52	*F. acuminatum* *F. armeniacum* *F. equiseti* *F. langsethiae* *F. poae* *F. sambucinum* *F. sporotrichioides*	[26,33,36,37]
Deoxynivalenol	DON	296.31	*F. boothii* *F. culmorum* *F. graminearum* *F. meridionale*	[26,32,33,36,37]
Nivalenol	NIV	312.32	*F. cerealis* *F. cortaderiae* *F. culmorum* *F. equiseti* *F. graminearum* *F. meridionale* *F. poae*	[26,32,33,36,37,39]
15-acetyl-deoxynivalenol and 3-acetyldeoxynivalenol	15- and 3-AcDON	338.35	*F. culmorum* *F. graminearum* *F. meridionale*	[32,33,37]

**Table 2:** Selected modified toxins produced by *Fusarium*, characteristic of maize kernels.

Mycotoxin	Abbreviation	Molar Mass [g/mol]	Type of Modification	Source
Deoxynivalenol 3-β-D -glucoside	DON-3G	458.46	plant	[26,33,37]
Zearalenone-14-sulphate	ZEN-14S	415.46	fungi	[26,37]
α-Zearalenol	α-ZOL	320.39	fungi or/and plant	[33,37]
β-Zearalenol	β-ZOL	320.39	fungi or/and plant	[33,37]
Hydrolysed fumonisin B_1_	HFB_1_	405.61	fungi	[26,37,48]

**Table 3:** Selected “emerging” toxins produced by *Fusarium* fungi, identified in maize.

Mycotoxin	Abbreviation	Molar Mass [g/mol]	Produced by:	Source
Beauvericin	BEA	783.96	*F. acuminatum* *F. anthophilum* *F. armeniacum* *F. avenaceum* *F. dlamini* *F. equiseti* *F. globosum* *F. nygamai* *F. oxysporum* *F. proliferatum* *F. poae* *F. sambucinum* *F. semitectum* *F. sporotrichioides* *F. subglutianans* *F. temperatum* *F. tricinctum* *F. verticillioides*	[26,33,36,37]
Culmorin	CUL	238.37	*F. culmorum* *F. cerealis* *F. graminearum* *F. langsethiae* *F. poae* *F. sporotrichioides* *F. venenatum*	[26,36,37]
15Hydroxyculmorin	15h-CUL	254.36	*F. culmorum* *F. graminearum* *F. poae*	[26,37]
5Hydroxyculmorin	5h-CUL	254.36	*F. culmorum* *F. graminearum* *F. poae*	[26]
Monoacetoxyscirpenol	MAS	324.40	*F. acuminatum* *F. equiseti* *F. sporotrichioides* *F. poae*	[26,33,37]
Diacetoxyscirpenol	DAS	366.41	*F. acuminatum* *F. armeniacum* *F. boothii* *F. cerealis* *F. cortaderiae* *F. culmorum* *F. equiseti* *F. graminearum* *F. meridionale* *F. sambucinum* *F. semitectum* *F. sporotrichoides* *F. poae*	[26,33,36,37]
Neosolaniol	NEO	382.40	*F. acuminatum* *F. poae* *F. sambucinum*	[26,33,37]
Moniliformin	MON	120.04	*F. acuminatum* *F. avenaceum* *F. chlamydosporum* *F. culmorum* *F. dlamini* *F. equiseti* *F. fusariodes* *F. oxysporum* *F. proliferatum* *F. semitectum* *F. sporotrichioides* *F. subglutians* *F. temperatum* *F. thapsinum* *F. tricinctum*	[26,33,34,36,37]
Enniatin A	ENN A	681.90	*F. acuminatum* *F. avenaceum* *F. chlamydosporum* *F. culmorum* *F. moniliforme* *F. nivale* *F. oxysporum* *F. poae* *F. proliferatum* *F. roseumF. solani* *F. sporotrichioides* *F. tritinctum*	[26,36,37]
Enniatin A_1_	ENN A1	667.90		
Enniatin B	ENN B	639.82		
Enniatin B_1_	ENN B1	653.85		
Enniatin B2	ENN B2	625.80		
Aurofusarin	Au-FU	570.50	*F. acuminatum* *F. avenaceum* *F. cerealis* *F. culmorum* *F. graminearum* *F. poae* *F. sambucinum* *F. sporotrichiodes* *F. tricinctum*	[26,36,37]
Bikaverin	BKV	382.3	*F. antholphilum* *F. bulbigenum* *F. dlamini* *F. fujikuroi* *F. moniliforme* *F. napiformi* *F. nygamai* *F. oxysporum* *F. proliferatum* *F. solani* *F. subglutitans* *F. verticillioides*	[26,37]
Butenolide	BUT	156.14	*F. cerealis* *F. culmorum* *F. graminearum* *F. poae* *F. sporotrichioides* *F. verticillioides*	[26,36,37]
Equisetin	EQ	373.49	*F. equiseti* *F. semitectum*	[37]
Fusaric acid	FA	179.22	*F. cerealis* *F. oxysporum* *F. proliferatum* *F. solani* *F. sublutinans* *F. thapsinum* *F. verticillioides*	[26,36,37]
Siccanol	SCN	402.58	*F. graminearum*	[26,37]
Fusaproliferin	FUS	444.60	*F. fujikuroi* *F. globosum* *F. proliferatum* *F. subglutinans* *F. temperatum*	[33,36,37]

**Table 4:** Natural occurrence of free and modified mycotoxins in maize.

Mycotoxin	Positive Samples (%)	Min-Max [µg/kg]	Origin	References
FB_1_	121/123 (98%)	12.6–8908	South Africa	[26]
	40/79 (51%)	68–2453 *	Egypt	[57]
	9/12 (75%)	<LOQ–49 **	China	[58]
	34/45 (76%)	59.9–9873	Albania	[59]
	79/90 (88%)	<LOD–45,145.82	Michigan (USA)	[60]
	70 (71.4%)	1080 ***	Spain	[61]
	48/55 (88%)	101–1838	West Africa (Togo)	[62]
	55/158 (34.8%)	60–553 *	Europe (various countries)	[63] ****
FB_2_	112/123 (91%)	7.9–3383	South Africa	[26]
	14/79 (18%)	4.7–386 *	Egypt	[57]
	27/45 (60%)	105–9218	Albania	[59]
	0/12 (0%)	<LOD	China	[58]
	76/90 (84%)	<LOD–22,538.63	Michigan (USA)	[60]
	55/98 (56.1%)	1306 ***	Spain	[61]
	30/55 (55%)	45–586.4	West Africa (Togo)	[62]
	46/158 (89.1%)	20.4–133 *	Europe (various countries)	[63] ****
FB_3_	98/123 (80%)	<LOQ–990	South Africa	[26]
	6/79 (8%)	16.8–286 *	Egypt	[57]
	72/90 (80%)	<LOD–17,972.72	Michigan (USA)	[60]
	14/55 (26%)	43–185.6	West Africa (Togo)	[62]
FB_4_	101/123 (82%)	<LOQ–1014	South Africa	[26]
	29/30 (96%)	461–2716 *	Nigeria	[64]
FB_1_ + FB_2_	148/148 (100%)	62.4–6	Brasil	[65]
		6274		
	34/45 (76%)	59.9–16,970	Albania	[59]
FA_1_	66/123 (54%)	<LOQ–51.4	South Africa	[26]
HFB_1_ *****	9/12 (75%)	144–7226 **	China	[58]
HFB_2_ *****	0/12 (0%)	<LOD	China	[58]
ZEN	41/123 (33%)	<LOQ–146	South Africa	[26]
	5/6 (83%)	<LOD–1071	Belgium	[46]
	10/79 (13%)	3.4–184 *	Egypt	[57]
	107/158 (67.7%)	15.2–1670 *	Europe (various countries)	[63] ****
	2/45 (4.4%)	218–263	Albania	[59]
	76/90 (84%)	0.56–4148.75	Michigan (USA)	[60]
	24/98 (24.5%)	110.3 ***	Spain	[61]
	1/55 (2%)	79–79	West Africa (Togo)	[62]
	115/120 (96%)	<LOD–3910	Germany	[66]
	42 (73.6%)	1.4–444.6	Brasil	[65]
	13/23 (56.52%)	<LOD–163.58	China	[52]
ZEN-14Glc	1/6 (17%)	274–274	Belgium	[46]
ZEN-14S	1/6 (17%)	51–51	Belgium	[46]
α-ZEL	6/6 (100%)	22–262	Belgium	[46]
	71/120 (59%)	<LOD–423	Germany	[66]
β-ZEL	4/6 (67%)	12–103	Belgium	[46]
	38/120 (32%)	<LOD–203	Germany	[66]
DON	61/123 (50%)	8.2–1380	South Africa	[26]
	11/150(7.3%)	270–1980	Ethiopia	[67]
	6/6 (100%)	411–5245	Belgium	[46]
	6/79 (8%)	311–807 *	Egypt	[57]
	11/45 (24%)	110–798	Albania	[59]
	120/120 (100%)	<LOQ–10,972	Germany	[66]
	107/158 (67.7%)	303–3060 *	Europe (various countries)	[63] ****
	87/90 (97%)	<LOD–20,475	Michigan (USA)	[60]
	81/81 (100%)	44–614	Northen Italy (Pidemont)	[68]
	63/81 (78%)	<LOD–216	Northen Italy (Lombardy)	
	72/81 (89%)	<LOD–83	Northen Italy (Vento)	
	31/98 (31.6%)	180 ***	Spain	[61]
	33 (58%)	41.6–1008	Brasil	[65]
DON-3G	65/123 (53%)	2.43–112	South Africa	[26]
	120/120 (100%)	95–3038	Germany	[66]
	4/79 (5%)	<LOQ–47.5 *	Egypt	[57]
	8/10 (80%)	14–121	China	[69]
	78/90 (87%)	<LOD–6266.49	Michigan (USA)	[60]
	40/158 (25.5%)	17.1–129 *	Europe (various countries)	[63] ****
3-AcDON	6/6 (100%)	63–643	Belgium	[46]
	29/90 (32%)	<LOD–63.04	Michigan (USA)	[60]
	5/98 (5.1%)	63.9 ***	Spain	[61]
	2/79 (3%)	<LOQ	Egypt	[57]
	108/257 (42%)	0–1046.8 *	Belgium	[70]
15-AcDON	6/6 (100%)	61–792	Belgium	[46]
	66/90 (73.3%)	<LOD–1787.6	Michigan (USA)	[60]
	112/257 (43%)	0–819.3 *	Belgium	[70]
NIV	14/123 (11%)	7.7–35.7	South Africa	[26]
	10/30 (33.3%)	5.1–50.8 *	Nigeria	[64]
	8/79 (10%)	1.6–142 *	Egypt	[57]
	10/30 (33.3%)	5.1–50.8	Nigeria	[64]
T-2	1/123 (0.8%)	148–148	South Africa	[26]
	1/45 (2.2%)	106–106 *	Albania	[59]
	17/90 (19%)	<LOD–156.65	Michigan (USA)	[60]
	5/98 (5.1%)	8.6 ***	Spain	[61]
	6/158 (3.8%)	2.55–4.08 *	Europe (various countries)	[63] ****
HT-2 toxin	1/123 (0.8%)	40.2–40.2	South Africa	[26]
	5/98 (5.1%)	11.7 ***	Spain	[61]
	4/90 (4%)	<LOD–276.74	Michigan (USA)	[60]

* median-maximum, no data about minimum content, ** values for fresh maize samples [58], *** median, **** maize silage [63], ***** hydrolysed fumonisin B_1_ and B_2_.

**Table 5:** Natural occurrence of emerging mycotoxins in maize.

Mycotoxin	Positive Samples (%)	Min–Max [µg/kg]	Origin	References
MON	120/123 (98%)	<LOQ–1130	South Africa	[26]
	26/29 (89.7%)	15.3–1450	Serbia (South-Backa)	[54]
	21/21 (100%)	5.06–850	Serbia (South-Banat)	
	3/6 (50%)	34.8–405	Serbia (West-Backa)	
	12/12 (100%)	7.18–1228	Serbia (Middle-Banat)	
	5/5 (100%)	3.03–3856	Serbia (Srem)	
	18/79 (23%)	1.6–142 *	Egypt	[57]
	54/90 (60%)	<LOD–1160.35	Michigan (USA)	[60]
	81/81 (100%)	93–751	Northen Italy (Pidemont)	[68]
	81/81 (100%)	592–4800	Northen Italy (Lombardy)	
	81/81 (100%)	8–1613	Northen Italy (Vento)	
	20/30 (66%)	25.3–1387 *	Nigeria	[64]
BEA	107/123 (87%)	<LOQ–142	South Africa	[26]
	20/21 (95.2%)	0.41–129	Serbia (South-Banat)	[54]
	26/29 (89.7%)	0.10–111	Serbia (South-Backa)	
	11/12 (91.7%)	0.23–49.7	Serbia (Middle-Banat)	
	4/5 (80%)	0.27–136	Serbia (Srem)	
	3/6 (50%)	0.03–18.2	Serbia (West-Backa)	
	30/30 (100%)	2.5–329 *	Nigeria	[64]
	80/90 (89%)	1.04–7446.21	Michigan (USA)	[60]
	50/79 (63%)	0.64–72 *	Egypt	[57]
FUS	16/21 (76.2%)	85.4–1121	Serbia (South-Banat)	[54]
	11/12 (91.7%)	450–1738	Serbia (Middle-Banat)	
	3/5 (60%)	312–4488	Serbia (Srem)	
	22/29 (75.9%)	91.3–4687	Serbia (South-Backa)	
	1/6 (16.7%)	12,272–12,272	Serbia (West-Backa)	
	2/30 (7.1%)	0.3–1.3	Nigeria	[64]
DAS	2/123 (1.7%)	4.4–5.0	South Africa	[26]
	9/55 (17%)	2.2–3	West Africa (Togo)	[62]
	12/30 (40%)	5.9–6.59 *	Nigeria	[64]
	22/257 (8.6%)	0–14.9 *	Belgium	[70]
MAS	1/123 (0.8%)	20.9–20.9	South Africa	[26]
	4/158 (2.5%)	9.91–32.9 *	Europe (various countries)	[63] **
NEO	1/123 (0.8%)	4.5–4.5	South Africa	[26]
ENN A	3/29 (10.3%)	0.12–0.47	Serbia (South-Backa)	[54]
	2/12 (16.7%)	0.41–17.1	Serbia (Middle-Banat)	
	1/6 (16.7%)	0.49	Serbia (West-Backa)	
	50/90 (56%)	<LOD–21.84	Michigan (USA)	[60]
ENN A1	4/90 (4%)	<LOD–27.28	Michigan (USA)	[60]
	3/29 (10.3%)	0.13–0.44	Serbia (South-Backa)	[54]
	3/12 (4.8%)	0.11–27.4	Serbia (Middle-Banat)	
	1/21 (4.8%)	0.59	Serbia (South-Banat)	
ENN B	47/90 (52%)	<LOD–2.34	Michigan (USA)	[60]
	1/21 (4.8%)	7.55	Serbia (South-Banat)	[54]
	2/12 (16.7%)	0.08–1.52	Serbia (Middle-Banat)	
	93/257 (36.2%)	46.2–1984.9 *	Belgium	[70]
ENN B1	6/90 (7%)	<LOD–7.94	Michigan (USA)	[60]
	1/21 (4.8%)	4.89	Serbia (South-Banat)	[54]
	1/6 (16.7%)	0.22	Serbia (West-Backa)	
	2/12 (16.7%)	0.20–16.3	Serbia (Middle-Banat)	
CUL	18/123 (15%)	13.3–465	South Africa	[26]
	125/158 (79.1%)	190–6680	Europe (various countries)	[63] **
BUT	35/123 (28%)	<LOQ–214	South Africa	[26]
	30/158 (19%)	28.9–583	Europe (various countries)	[63]
FA	24/123 (20%)	57–195	South Africa	[26]
	35//158 (22.2%)	229–4120	Europe (various countries)	[63]
Bikaverin	82/123 (67%)	<LOQ–651	South Africa	[26]
	42/158 (26.6%)	20.3–415	Europe (various countries)	[63] **
15Hydroxyculmorin	49/123 (40%)	<LOQ–2022	South Africa	[26]
	84/158 (53.2%)	229–1670	Europe (various countries)	[63]
5Hydroxyculmorin	18/123 (15%)	<LOQ–578	South Africa	[26]
	19/158 (12%)	571–1480	Europe (various countries)	[63] **
Apicidin	2/123 (1.6%)	2.9–15.4	South Africa	[26]
	79/123 (50%)	9.49–175	Europe (various countries)	[63] **
Aurofusarin	89/123 (72%)	<LOQ–5470	South Africa	[26]
Epiequisetin	19/123 (15%)	<LOQ–18.9		
Equisetin	30/123 (24%)	<LOQ–129		
Fusarinolic acid	24/123 (20%)	<LOQ–3422		
Acuminatum B	12/123 (9.8%)	<LOQ–219		
Acuminatum C	7/123 (5.7%)	<LOQ–204		
Chlamydospordiol	2/123 (1.7%)	2.1–5.1		
Chlamydosporol	1/123 (0.8%)	87.0–26.9		
Chrysogin	48/123 (30%)	<LOQ–7.7		
Siccanol	91/123 (74%)	34.6–252		
Fusapyron	38/123 (31%)	<LOQ–18.0		

Median-maximum, * no data about minimum content, ** maize silage [63].

## Data Availability

Not applicable.
